# Surgery

**Published:** 1877-03

**Authors:** 


### II. Surgery.

1. *Inflammatory and Necrotic Processes in Bone.* Busch. (*Langenbeck's Arch. XX.; N. Y. Med. Record.*)

The various stages through which bone passes from inflammation to total necrosis have been carefully studied by Professor Busch, of Berlin, in a series of experiments on animals; and his results throw some light upon these rather obscure processes. His plan was to bore into the medullary canal of a long bone, near the articular extremity, then pass a wire of platinum or iron along the cavity, bringing it out again through a second opening near the opposite extremity. The wire was then heated to whiteness by the galvano-caustic battery. In a large number of cases various degrees of damage were done to the bone; in some, however, where the platinum wire had been allowed to remain in the bone, no apparent reaction was produced, while the holes through which it had entered were closed. These results tally with those published by Cruveilhier in 1816. Necrosis, the author believes, does not in such cases depend upon the mere presence of a foreign body, but upon some of the physical qualities that are associated with it. The expansion of laminaria, when introduced into the medullary cavity, determines the necrosis; and so with platinum or iron, the chemical or other qualities associated with it are the real exciting agents. Four grades of inflammation, corresponding to the intensity of the cause, were recognized: 1, active inflammation (ostitis); 2, death of a greater or less extensive lamella from the inner surface of the compact substance (necrosis interna seu centralis); 3, death of the entire thickness of the compact cortex (penetrating necrosis), or of the whole circumference of the bone (total necrosis); finally, 4, death of the entire bone, with perhaps some of the enveloping soft parts.

The first-named results were observed in three dogs, the examinations having been made on the 53d, 67th, and 73d days after the injury. The author also adds, as an interesting fact, that where necrosis was observed in one of the principal bones of the limb, it was not uncommon to find that the adjacent bone was affected with ostitis. The various stages of the inflammatory process in a bone were found to follow each other in the following order: Swelling and thickening of the periosteum; deposit of bone substance on the outer surface of the cortex; formation of bone in the medullary cavity; rarefaction of the tissue of the old compact tissue (enlargement of the Haversian canals, and with or without implications of the lacunæ), so that it looked precisely like the new bony matter; thickening of the outermost layers of the periosteal deposits, by which a certain sort of new bony cortex was formed; finally, there was an apparent compression of the inner bony structures by connective tissue, so that in this way there was a new central cavity partly filled with fibrous tissue.

In the cases in which the second grade of the process was observed, it was about the holes that had been bored. In the remainder of the bone there was ostitis. The sequestrum was not fully loose, the examination being made at times ranging from the 30th to the 57th day. The sequestrum was either tubular, or at any rate curved. There was in this case also a deposit of new bone beneath the periosteum, the direction of the new bony fibers being vertical to the surface of the bone. The thickness of the deposit was greatest over the center of the bone. The separation of the sequestrum was caused by the development of granulations in the interior of the old compact substance, while its remaining portion, together with the new deposit, formed the involucrum. In the third grade, where the entire thickness of the bony substance was destroyed, the sequestrum had a smooth surface, like a macerated bone. A point clearly shown in this class of cases was, that, wherever the outer surface of the sequestrum was smooth, there was a corresponding opening in the bony capsule. In the two cases of total necrosis, the involucrum consisted of two parts, the support of the limb being maintained by the fibula. This fact is said to agree with the experiences of Scarpa, Sédillot, and others, that, where the entire thickness of the compact substance takes place, there is no regeneration of bone tissue. It is to be explained by the fact that in these cases the periosteum is separated from the bone, which sets up suppuration, and thus bone formation is prevented. In total necrosis the continuity of the bony involucrum is endangered; in penetrating necrosis a continuous involucrum remains. The early or late removal of the sequestrum appeared to have no influence on the formation of bone. In applying these results to the question of operative surgery in children and adults, the author believes that much the same consequences may be expected. In children, where there is total necrosis of a portion of the bone, if there is bony union, shortening may be expected; but he thinks that after the twenty-fifth year the cases are very rare in which bony substitution occurs after total necrosis.

Of the fourth class, where death of the entire bone took place, the animals all died at times between the end of the first day and of the first week. There was no reaction in any of them.

2. *Two Cases of Ascites Successfully Treated with Iodine Injections.* NORWOOD. (*The Canada Lancet*, February, 1877.)

Sixty-seven operations of paracentesis abdominis, upon a gentleman sixty-six years of age, during a period of eighteen months, with the removal of from eighteen to twenty-four quarts of fluid at each sitting, were made prior to iodine treatment. About half the usual amount of fluid being removed, as a preparatory step to the operation, two ounces of tinct. iodin. co., diluted with an equal quantity of distilled water, were injected into the cavity of the peritoneum; cannula was plugged, and patient rolled gently over on the bed for about ten minutes, to bring the injected fluid in contact with the whole surface of the sac. The plug remaining a half hour, was removed, and the balance of fluid (six quarts) drawn off, and a bandage applied. The day following the operation the patient had a chill, with some febrile excitement, and tenderness all over the abdomen. He was kept profoundly under the influence of opium, and had three-grain doses of calomel every four hours. He soon began to convalesce, and in about three weeks from the operation (during which time he received three small tappings) was discharged cured. W. F. L.

3. *Case of Transverse Fracture of the Patella, Treated by a New Method.* GRANT. (*Edinburgh Med. Jour.*, October, 1876.)

After an interval of two days, to let the swelling subside a little, Dr. G. treats transverse fractures of the patella as follows: The leg is placed on an inclined splint, extending from heel to near gluteal fold; the lower fragment firmly fixed in position by a strap of plaster passing around the leg, and a semilunar splint of Hyde's poroplastic material carefully modeled to the thigh just above the margin of the upper fragment, and is held in position by two stout pieces of strapping, the whole being surrounded by a few turns of a convergent spica bandage. The splint is then allowed to "set," and two steel hooks (the size is not mentioned) are fixed firmly into the splint, one on each side of the patella. The hooks are connected with a steel chain about three feet long, and this attached to the ordinary pulley extension apparatus with a weight of nearly four pounds; this brings the upper fragment down, while the lower remains in position. The method is painless and efficient. W. F. L.

4. *Extirpation of a large Lipoma of a Bleeder.* STILLING. (*Deutsche Med Wochenschr.* 51, 1876.)

Mr. Sp., aged fifty-eight years, has had a large lipoma on his back for a great many years. As long as it did not cause him any great inconvenience he would not have anything done for it, especially as he knew in his youth to have bled fearfully after the slightest injury, such as a puncture with a pin. In 1876, however, the tumor had reached the size of a child's head, and the patient submitted to the extirpation. The operation was performed under the carbolized spray; in fact, Lister's antiseptic method was strictly, faithfully adhered to.

The bleeding was exceedingly severe, and very many arteries, though small, had to be closed by a cat-gut ligature or by torsion. The large wound in the skin being closed by numerous cat-gut sutures, a pressure bandage of salicylated cotton was applied. Half an hour after the patient was dressed blood began oozing out from under the bandage, and continued doing so during a whole night. The next morning, when the dressing was changed, the borders of the wound were united, but underneath the effused blood had accumulated in such a quantity as to form a tumor as large as the original lipoma; the tumor showed decided fluctuation—the blood was not coagulated yet. On the fourth day the wound was completely healed by first union; the hæmorrhagic tumor was solid, and under the same dressing, continued during four weeks, it was entirely re-absorbed. During all this time the patient's pulse never rose above eighty; the temperature was always normal; no fever, nor any secretion of pus.

5. *Excision of the Infraorbital Nerve; Rapid Recovery under the Antiseptic Treatment.* Stilling. (*Deutsche Med. Wochenschr.* 52, 1876.)

Mrs. R., aged 72 years, suffered from facial neuralgia since July, 1876. The first attack set in while she was washing her face; it lasted but one minute. Afterward the attacks were occasioned also by speaking, eating and deglutition. The neuralgia was the most violent in the region of the infraorbital nerve, though it extended also over the ramifications of the dental, zygomatic, inferior maxillary nerves. No cause could be ascertained; no relief from general treatment.

October 21, the entire piece of the infraorbital nerve contained in the infraorbital canal was removed by the usual *modus operandi.* The operation was performed under the carbolized spray, and the wound was dressed scrupulously after Lister. The first dressing was not changed till the sixth day; the wounds had healed by first union; not one single drop of pus had been secreted.

On the eleventh day the patient could be discharged as cured. The neuralgic pains had not returned in the zygomatic and the other branches after the excision of the infraorbital nerve.

